# Unilateral gingival fibromatosis with localized aggressive periodontitis (involving first molars): An unusual case report

**DOI:** 10.4103/0972-124X.55834

**Published:** 2009

**Authors:** Sharn Pal Sandhu, Vipin Kakar, Guneet Gogia, S. C. Narula

**Affiliations:** *Professor and Head, Department of Periodontics and Oral Implantology, S.G.T. Dental College, Budhera, Gurgaon*; 1 *Associate Professor, Department of Periodontics and Oral Implantology, S.G.T. Dental College, Budhera, Gurgaon*; 2 *Sr. Lecturer, Department of Periodontics and Oral Implantology, S.G.T. Dental College, Budhera, Gurgaon*; 3 *Professor and Head, G D C Rohtak, Haryana, India*

**Keywords:** Aggressive periodontitis, fibrocellular proliferation, gingival fibromatosis, puberty onset, vertical bone loss around first molars

## Abstract

An atypical and rare case report is presented here of a 16 years old female patient who presented with severe, unilateral, gingival enlargement along with aggressive periodontitis around first molars that was confined to the left side of her mouth. A careful recording of the case history and results of clinical examination, laboratory blood analysis, radiological findings, and microbiological and histopathological investigations were noted and a critical review of similar conditions was taken into account to arrive at the said diagnosis.

## INTRODUCTION

Among the various types of gingival enlargements, gingival fibromatosis presents with features that are distinguishable from other types of gingival enlargements, but, at the same time, the presentation of the clinical features is quite varied.

Clinical gingival fibromatosis is a slowly progressive, gingival enlargement caused by the increased production of collagen in the lamina propria of the gingiva. The enlargement generally begins before the age of 20 years and is often correlated to the eruption of deciduous or permanent teeth.[1] Several investigators believe that the presence of teeth is a precondition for gingival fibromatosis to occur. The enlargement gradually increases in size and may even overgrow the associated teeth and interfere with normal function, even lip closure.[1] If gingival fibromatosis is associated with an erupting tooth, it may even prevent the visualization of the tooth even after it has fully erupted.[2]

The enlargement may be associated with one or more teeth, involve one or more quadrant(s), or may be generalized. The lingual or palatal gingivae are typically increased in thickness when compared to the bucccal side. In the localized form, gingival fibromatosis may remain dormant and may suddenly extend to involve other segments of one or both jaws. One distinctive but not uncommon pattern involves the posterior maxillary alveolar ridge. This is usually seen to be bilaterally symmetrical, extending posteriorly and palatally from the posterior alveolar ridges.

The etiology of gingival fibromatosis is thought to be familial or idiopathic. The familial variation may occur as an isolated finding or be associated to one of several hereditary syndromes, *e.g*., Zimmermann-Laband, Murray-Puretic-Drescher, Rutherfurd, multiple hematoma, and Cross syndrome.[3] The exact cause of this abnormality is unknown, but it is thought to be an autosomal dominant pattern of inheritance in most cases. However, autosomal recessive examples have also been noted.[1]

Among all the forms of periodontitis, aggressive periodontitis has received considerable attention due to its peculiar clinical presentation: occurring around puberty with an apparent lack of local factors such as heavy amounts of plaque and calculus in patients with reasonably good oral hygiene.

The disease appears to be the result of a defect in the immune response rather than plaque and calculus deposition.[2] It has been shown by many investigators that patients with aggressive periodontitis display functional defects of PMNL, monocytes or both, but without any systemic manifestations.[4] This results in a reduced defensive ability against some of the periodontal pathogens. Aggressive periodontitis has a familial tendency which suggests a genetic predisposition.[4]

The localized form of aggressive periodontitis predominantly affects the 1^st^ molar and the incisors with loss of attachment in at least two permanent teeth, one of which is the 1^st^ molar. The rate of alveolar bone loss is considerably higher in aggressive periodontitis than in chronic periodontitis.[4] A striking feature is the absence of clinical inflammation with minimal local factors despite the presence of a deep periodontal pocket. Various periodontal pathogens have been implicated in sites of aggressive periodontitis, but the role of *Actinobacillus actinomycetemcomitans* has been the predominant one.

Radiographically, there is a characteristic vertical loss of alveolar bone around the 1^st^ molar and incisors at around the age of puberty in an otherwise (apparently) healthy teenager. Several authors have referred to it as an arc-shaped bone loss which extends from the distal surface of the 2^nd^ premolar to the mesial surface of the 2^nd^ molar.[4]

As presented here, the clinical features of localized forms of aggressive periodontitis can be varied. Such cases of aggressive destruction of the attachment apparatus are unfortunately misinterpreted as some other entity due to the presence of some other divulging clinical or systemic feature.

## CASE REPORT

A 16 year-old girl presented in the Department of Periodontics and Oral Implantology at the SGT Dental College, Village Budhera, Gurgaon (Haryana), with the chief complaint of gingival growth/swelling around the left upper and left lower first molars and an inability to chew food on the left side.

Her case history revealed that the swelling first appeared two years ago with no associated symptoms and gradually increased to its present size. The patient came for treatment only when the swelling started interfering with mastication. There was no family history and no relevant medical history in the patient. Upon general physical examination, she was found to have pallor of conjunctiva and oral tissues and appeared anemic.

**Figure 1:**
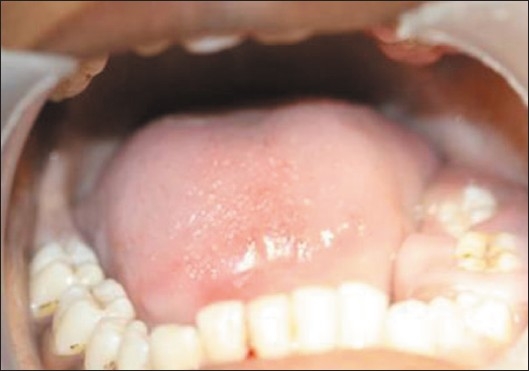
Gingival hyperplasia on the left side of mandibular arch

**Figure 2:**
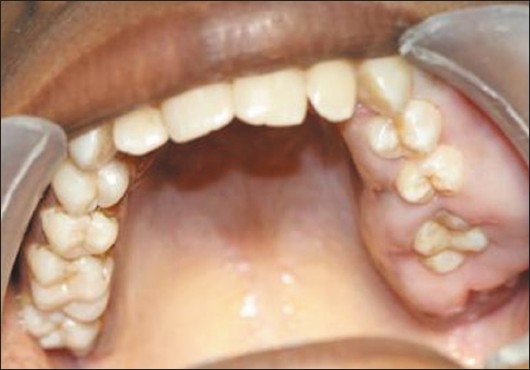
Gingival hyperplasia on the left side of maxillary arch

**Figure 3:**
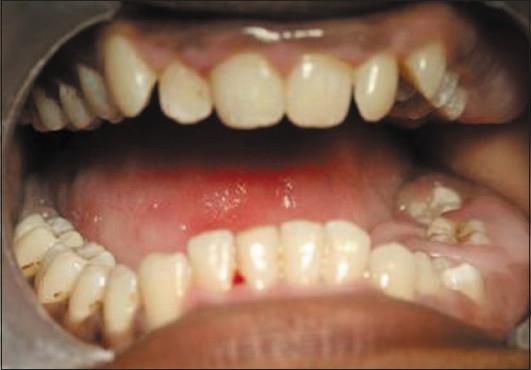
Gingival enlargement up to the occlusal surfaces, submerging the teeth

Upon intraoral examination, gingival hyperplasia was found around the first molars on the left side, extending significantly around the adjacent teeth, both in the upper arch [Figures 1 and 2] and the lower arch [Figures 3]. The right side of the mouth was unaffected. The enlarged gingiva was smooth but firm, exhibited no color change/altered surface characteristics and extended up to the occlusal line angles of the teeth.

**Figure 4:**
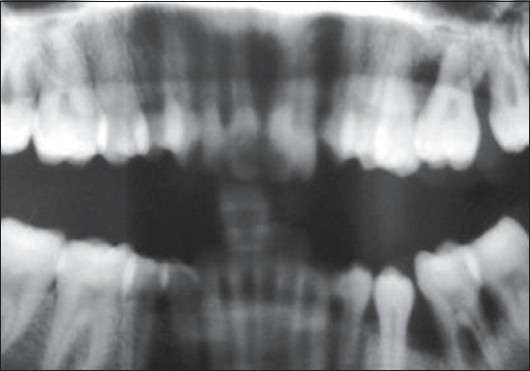
Panoramic radiograph

**Figure 5:**
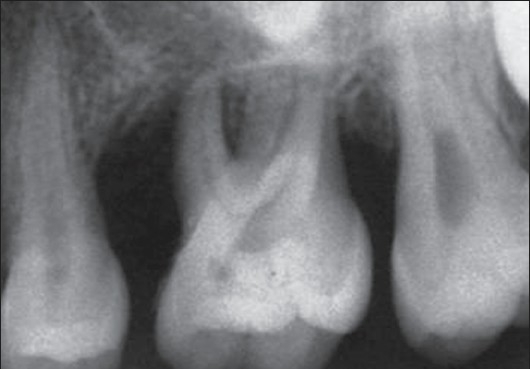
IOPA of maxillary left first molar (26)

**Figure 6:**
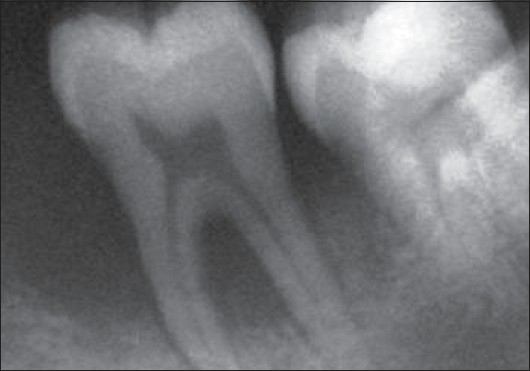
IOPA of mandibular left first molar (36)

**Figure 7:**
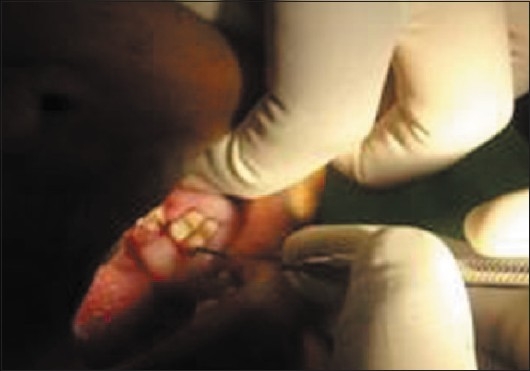
Deep periodontal pocket around 36 (Mesiolingual aspect)

**Figure 8:**
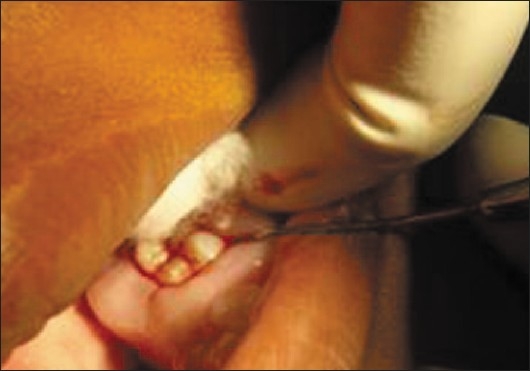
Deep periodontal pocket around 36 (mesiobuccal aspect)

**Figure 9:**
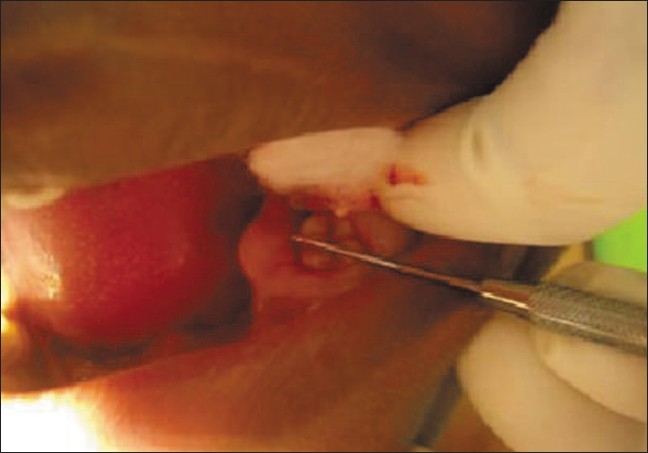
Deep periodontal pocket around 36 (midlingual aspect)

The pockets were 10–14 mm deep [Figures 4–6] with an attachment loss of 5–7 mm; the mobility was grade 2 around the mandibular 1^st^ molar and the maxillary 1^st^ molar, and grade 1 around the mandibular 2^nd^ molar [Figures 7–9].

There was no significant pain for the patient and probing revealed little subgingival plaque and calulus.

### Macroscopic examination of gingiva

Gingival enlargement was restricted to the left side of the mouth with involvement of both the upper as well as lower arches [Figures 1 and 2]. The enlargement seemed to be progressing around the 2^nd^ molars to the incisors, although the molars showed maximal growth. The enlargement involved the attached gingiva as well as the gingival margin and the interdental papillae. Both facial and lingual/palatal gingivae were affected.

The enlarged gingiva was pale pink and firm, but not fibrotic. The teeth were almost completely covered with gingival tissue on all sides, except for the occlusal surface [Figure 3]. The jaws appeared distorted because of the bulbous enlargement of the gingiva. No secondary inflammation was observed at the gingival margin.

### Haematological investigations

Routine hematological investigations revealed a decreased hemoglobin count of 5.3 g% and a differential leukocyte count of polymorphonuclear leukocytes (neutrophils) 58%, lymphocytes 38%, monocytes 2%, eosinophils 2%, and a random blood sugar level of 98 mg/dl.

### Radiological examination

Radiographic examination (OPG and IOPA X-rays) revealed an arc-shaped bone loss around the 1^st^ molars on the left side, with the right side remaining unaffected [Figures 4–6].

### Microbiological examination

**Figure 10:**
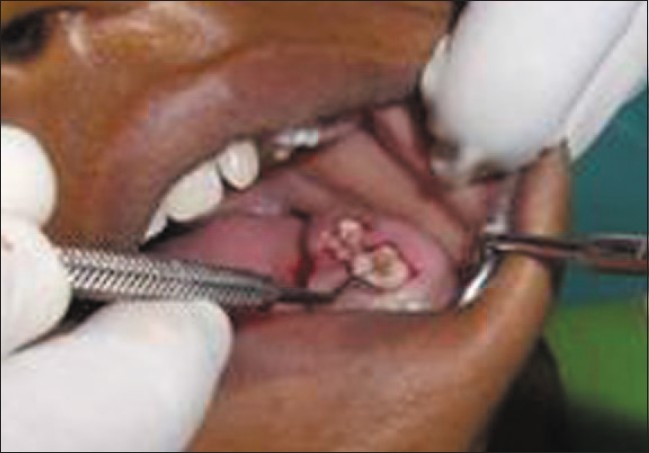
Subgingival plaque taken for culture

The patient's subgingival dental plaque was taken for anaerobic culture in meat broth medium [Figure 10]; no anaerobic growth was seen.

### Biopsy

As the patient was severely anemic, she was sent for a medical consultation to determine whether a biopsy could be taken. After obtaining the physician's clearance, an incisional biopsy of the gingival tissue was obtained under local anesthesia utilizing a reverse bevel flap incision. There was a significant absence of hard deposits and scanty dental plaque was seen upon elevation of the flap.

**Figure 11:**
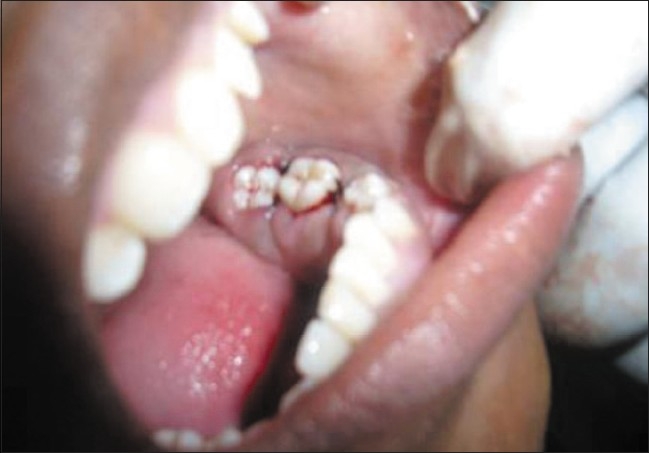
Sutures given after taking biopsy

Sutures were given [Figure 11] and the patient was put on prophylactic antibiotics and anti-inflammatory agents for three days along with hexidine mouthwash. Diet instructions were given and patient was recalled after one week.

### Histopathological features

**Figure 12:**
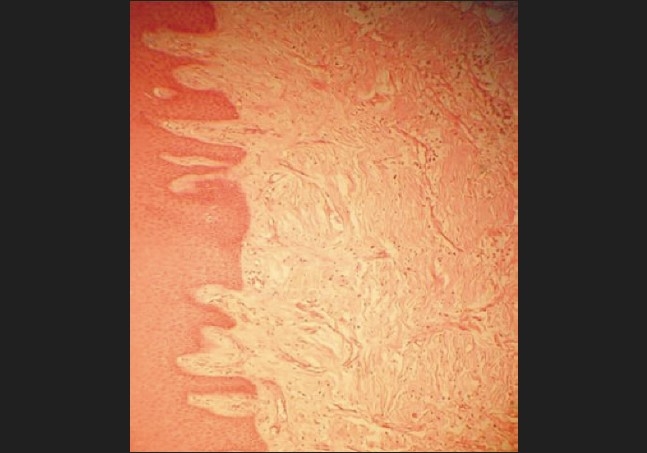
Histopathological picture of the biopsy specimen

Histopathological examination showed an increase in the amount of connective tissue that was relatively avascular and consisted of increased fibrocellular content. There were numerous immature collagen fibres with abundant fibroblasts. The surface epithelium was acanthotic and parakeratotic [Figure 12]; no giant cells were present. The gingival picture clinically simulated idiopathic gingival hyperplasia that was restricted to the left side of the mouth only.

**Figure 13:**
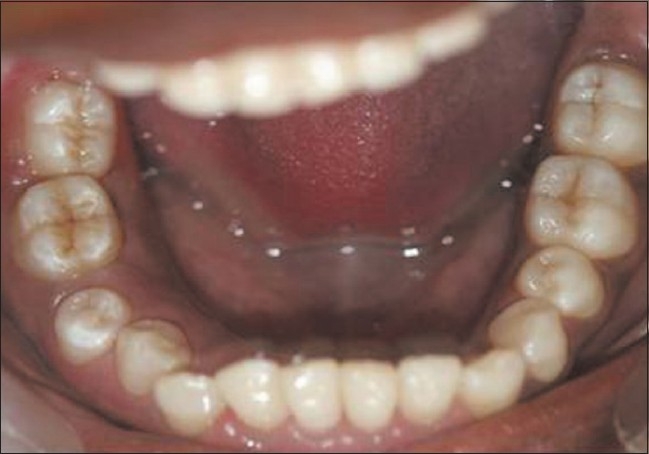
Postoperative view (mandibular)

**Figure 14:**
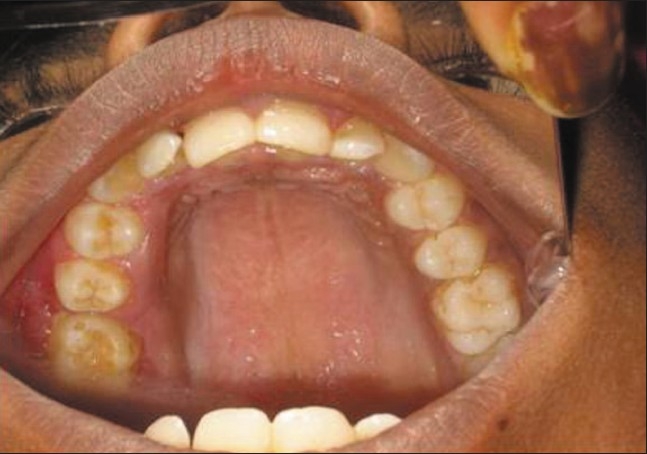
Postoperative view (maxillary)

Healing was neventful; sutures were removed after one week [Figures 13 and 14].

## DISCUSSION

The patient gave a history of the onset of her condition at around puberty.[5] Clinically, the patient had characterized “first molar” presentation with interproximal attachment loss on the two permanent teeth, the left upper and left lower molars. The pattern of alveolar bone loss was “arcuate”, extending from the distal surface of the second premolar to the mesial surface of the second molar, both in the upper as well as the lower jaws on the left side. There was a lack of clinical inflammation despite the presence of deep periodontal pockets and advanced bone loss. The amount of plaque on the affected teeth was minimal, which seemed inconsistent with the amount of periodontal destruction present. The facts that the patient is a female and the onset had been circumpubertal also supported the clinical picture of localized aggressive periodontitis.[6]

Also co-existent was the clinical picture of gingival fibromatosis corroborated by the histopathologically characteristic fibrocellular proliferation seen in idiopathic gingival hyperplasia.[7]

This case is thus a rare and atypical presentation of a true combined lesion of gingival fibromatosis with localized aggressive bone destruction, which is not a common feature seen in gingival fibromatosis. The involvement of the first molars and the typical arcuate bone loss pattern at the given age of the patient, suggest localized aggressive periodontitis. It is difficult to retrace the clinical presentation and identify which of the two occurred first, but what is important is that the patient is predisposed to both the pathological processes which have aggravated each other, even in the absence of any other predisposing local or systemic factors.
